# SGLT2i and GLP-1RA in Cardiometabolic and Renal Diseases: From Glycemic Control to Adipose Tissue Inflammation and Senescence

**DOI:** 10.1155/2021/9032378

**Published:** 2021-11-08

**Authors:** Luis D'Marco, Valery Morillo, José Luis Gorriz, María K. Suarez, Manuel Nava, Ángel Ortega, Heliana Parra, Nelson Villasmil, Joselyn Rojas-Quintero, Valmore Bermúdez

**Affiliations:** ^1^Hospital Clínico Universitario de Valencia, INCLIVA, Valencia 46010, Spain; ^2^CEU Cardenal Herrera University, Valencia 46115, Spain; ^3^Endocrine and Metabolic Diseases Research Center, School of Medicine, University of Zulia, Maracaibo 4004, Venezuela; ^4^School of Medicine, University of Zulia, Maracaibo 4004, Venezuela; ^5^Division of Pulmonary and Critical Care Medicine, Brigham and Women's Hospital, Harvard Medical School, Boston, MA 77054, USA; ^6^Universidad Simón Bolívar, Facultad de Ciencias de la Salud, Barranquilla 080002, Colombia

## Abstract

*Background*. Over the last few years, the use of sodium-glucose cotransporter 2 inhibitors (SGLT2i) and glucagon-like peptide 1 receptor agonists (GLP-1RA) has increased substantially in medical practice due to their documented benefits in cardiorenal and metabolic health. In this sense, and in addition to being used for glycemic control in diabetic patients, these drugs also have other favorable effects such as weight loss and lowering blood pressure, and more recently, they have been shown to have cardio and renoprotective effects with anti-inflammatory properties. Concerning the latter, the individual or associated use of these antihyperglycemic agents has been linked with a decrease in proinflammatory cytokines and with an improvement in the inflammatory profile in chronic endocrine-metabolic diseases. Hence, these drugs have been positioned as first-line therapy in the management of diabetes and its multiple comorbidities, such as obesity, which has been associated with persistent inflammatory states that induce dysfunction of the adipose tissue. Moreover, other frequent comorbidities in long-standing diabetic patients are chronic complications such as diabetic kidney disease, whose progression can be slowed by SGLT2i and/or GLP-1RA. The neuroendocrine and immunometabolism mechanisms underlying adipose tissue inflammation in individuals with diabetes and cardiometabolic and renal diseases are complex and not fully understood. *Summary*. This review intends to expose the probable molecular mechanisms and compile evidence of the synergistic or additive anti-inflammatory effects of SGLT2i and GLP-1RA and their potential impact on the management of patients with obesity and cardiorenal compromise.

## 1. Introduction

Type 2 diabetes mellitus (T2DM) is a growing global cause of morbidity and mortality, with 629 million people expected to suffer from this disease by 2045 [1]. T2DM is tightly associated with comorbidities such as obesity, hypertension, and dyslipidemia, as well as with a wide range of macrovascular and microvascular complications such as chronic kidney disease (CKD) and heart failure (HF) [2, 3]. Consequently, there is a constant demand for the development of drugs to control hyperglycemia and influence the management of T2DM comorbidities [4, 5].

Over the last few years, the use of sodium-glucose cotransporter 2 inhibitors (SGLT2i) and glucagon-like peptide 1 receptor agonists (GLP-1RA) has increased substantially in the medical practice. These drugs have changed the standpoint on the comprehensive management of diabetic patients, and their effects are not limited to glycemic control [6]. They help in this regard by inhibiting glucose reabsorption in the proximal tubule and by, their incretin effect, stimulating insulin secretion by binding to specific receptors in the pancreatic *β* cells [7, 8]. They have also proven their efficacy in fat loss, blood pressure reduction, effect on certain oncological diseases, and more recently, their cardio- and renoprotective anti-inflammatory effects [9, 10].

In this sense, their beneficial role in diabetic kidney disease (DKD) stands out since this disease's progression can be modified by the administration of these drugs [11–14]. There has also been a favorable response to these agents in the treatment of obesity, a comorbidity characterized by an excess or excessive accumulation of fat tissue. This is achieved via mechanisms that involve a decrease in appetite and the reduction of caloric intake, independently of their glucose-lowering effects [15, 16].

Regarding the anti-inflammatory effect, these drugs have been associated with a decrease in proinflammatory cytokines and oxidative stress. They also change the polarization of immune cells towards an anti-inflammatory role, and they decrease the recruitment and accumulation of T cells and M1 macrophages, especially in the adipose tissue and organ-specific fat pads such as perirenal and epicardial fat. Furthermore, it has been observed that they have a certain effect on the attenuation of cellular senescence [17], a process initiated in response to cell stress and damage observed in these patients. Hence, they could also be a therapeutic tool for managing inflammation and premature senescence of chronic endocrine-metabolic diseases [9].

The beneficial mechanisms of these drugs, besides their glucose-lowering effect, are complex and have not been completely understood. Therefore, this remains an area of fertile and active investigation. After reviewing the main findings and new evidence regarding these drugs, this review explores the possible molecular mechanisms by which these novel drugs affect the adipose tissues through antisenescent and anti-inflammatory properties and their potential impact on the management of patients with diabetes, obesity, and/or cardiorenal involvement.

## 2. SGLT2i: More Than Antidiabetic Drugs

SGLT2i, commonly known as gliflozins, constitute the most recent class of oral hypoglycemic agents (OHA) approved by the U.S. Food and Drug Administration (FDA) to treat T2DM. Other OHA work by improving insulin sensitivity and glucose uptake as well as by restoring beta cells; gliflozins have a different mechanism focused on the kidneys, specifically in the first portion of the proximal tubule (PT), where they act by inhibiting SGLT2 suppressing the reabsorption of glucose and increasing its urinary excretion [18].

The glycemia levels capable of triggering glycosuria are observed when serum glucose is approximately 180 mg/dL in euglycemic subjects or 200 mg/dL in patients with diabetes [19]. The explanation for this higher glycemic threshold in diabetic patients is still unknown. Some studies suggest that this may be due to the presence of maladaptive mechanisms, wherein hyperglycemia increases the expression and hyperactivity of the SGLT2 in the proximal tubule. This elicits an increase in the reabsorption of filtered glucose [20, 21]. SGLT2i drugs oppose these mechanisms and promote glycosuria, lowering serum glucose in patients with diabetes. All this occurs independently of insulin secretion, so the risk of hypoglycemia is minimal [22].

**Figure 1:**
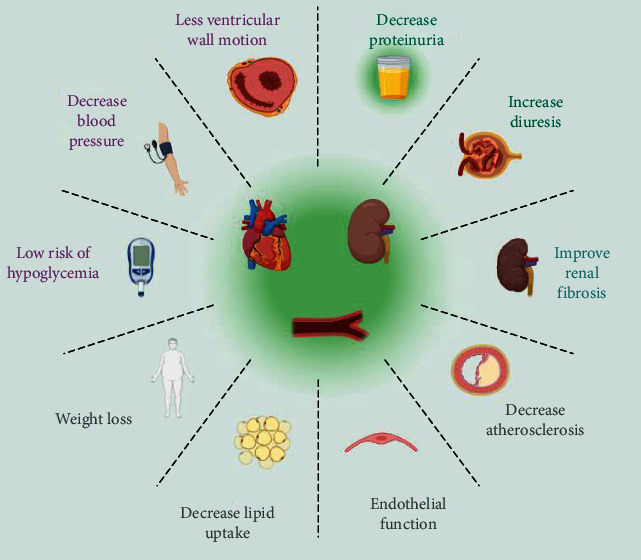
Proven benefits of SGLT2i and GLP-1RA in the cardiorenal-vascular axis. Strong evidence supports the multiple effects of SGLT2i and GLP-1RA on the cardiovascular disease scenario. Studies show cardio- and renoprotective properties in clinical biomarkers and long-term mortality. SGLT2i: sodium-glucose cotransporter 2 inhibitors; GLP-1RA: glucagon-like peptide 1 agonists.

Beyond this antidiabetic effect, SGLT2i drugs have reported benefits in cardiac and renal diseases (Figure 1). The DAPA-CKD trial has shown in patients with CKD, regardless of the presence or absence of diabetes, that the uses of dapagliflozin against placebo significantly improve the risk of decline in the eGFR at least in 50% of the patients, end-stage kidney disease, or death from renal or cardiovascular causes [23]. In patients with (HF) and reduced ejection fraction, the DAPA-HF trial reported that the risk of cardiac worsening or death from cardiovascular causes was lower among those patients who received dapagliflozin than placebo controls [24]. Similarly, in the EMPEROR-Reduced trial in patients receiving HF standard care therapy, those in the empagliflozin arm had a lower risk of hospitalization for HF or cardiovascular death than those in the placebo group, also regardless of the presence or absence of diabetes [25]. Designed in patients with HF, in the EMPEROR-Preserved trial, the annual rate of decline of eGFR was slower in the SGLT2i (empagliflozin) group than in the placebo, decreasing the risk of serious renal outcomes [25]. Other randomized trials and meta-analyses have shown similar benefits with SGLT2i in the cardiorenal field (Table 1) [26–29].

### 2.1. SGLT2i in Cardiometabolic and Renal Diseases

**Figure 2:**
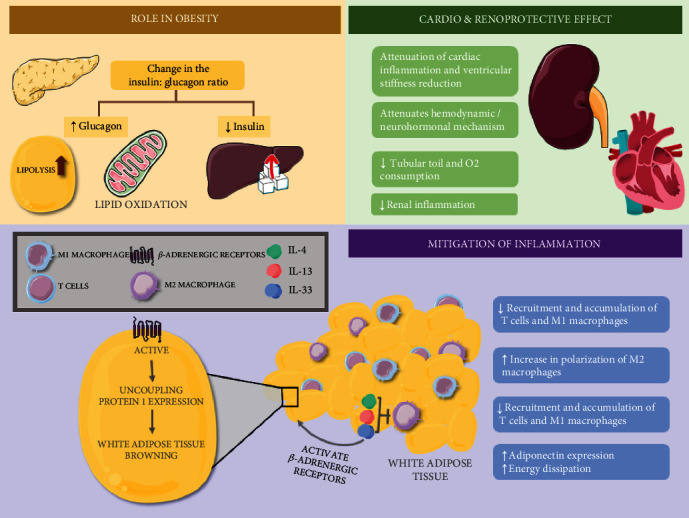
Role of SGLT2i in obesity, adipose tissue inflammation, and cardiac and kidney disease. In obesity, these drugs modify the insulin : glucagon ratio, where glucagon increases, favoring lipolysis and lipid oxidation, while insulin decreases, causing an increase in endogenous glucose production from amino acids, favoring the process of lipolysis even more. Also, they attenuate the hemodynamic/neurohormonal mechanism in the kidney with positive changes of glomerular filtration rate (GFR), the tubular transport toil, and oxygen consumption. Ultimately, they have a role in the mitigation of inflammation decreasing the recruitment and accumulation of T cells and M1 macrophages, increasing the polarization of M2 macrophages, which release anti-inflammatory cytokines while activating adrenergic receptors in adipocytes, producing thermogenesis by the expression of uncoupling protein (UCP1). Furthermore, they increase adiponectin expression, which promotes the downregulation of SGLT2 and subcutaneous white adipose tissue (WAT) browning by stimulating the proliferation of M2 macrophages. SGLT2i: sodium-glucose cotransporter 2 inhibitors; GFR: glomerular filtration rate; UCP: uncoupling proteins; WAT: white adipose tissue.

The hyperactivity of SGLT2 exerts direct renal effects on the PT, where the increase in the reabsorption of the filtered load of glucose and sodium leads to a decrease in the sodium supply and transport towards the macula densa, which causes decreased ATP degradation and adenosine production. When the latter drops, it promotes vasodilation of afferent arterioles, and therefore, a glomerular hyperfiltration followed by an increase in intraglomerular pressure [30]. This glomerular hyperfiltration increases the toil of the tubular transport, causing an increase in oxygen consumption and hypoxia and favoring the development of renal interstitial fibrosis [31]. The use of SGLT2i attenuates this hemodynamic/neurohormonal mechanism, not affecting the GFR in the long term, the toil of the tubular transport, and oxygen consumption, generating a renoprotective effect (Figure 2) [32].

On the other hand, studies performed on mice with induced DM have also demonstrated the role of SGLT2i in the decrease of kidney inflammation, showing a significant reduction in the renal expression of proinflammatory cytokines and chemokines such as tumor necrosis factor alpha (TNF-*α*) and the monocyte chemoattractant protein 1 (MCP-1), as well as urinary markers of renal inflammation (IL-6) and the suppression of apoptotic markers like the suppression of caspase-3. Furthermore, they have been associated with a lower expression of profibrotic genes, including the transforming growth factor beta (TGF*β*), type IV collagen, and fibronectin [33]. Similar results were reported in humans by Dekkers et al. [34], who evaluated the renoprotective effect of dapagliflozin, an SGLT2i, by determining tubular and inflammatory markers in patients with T2DM, reporting that compared to placebo, there is a decrease in urinary excretion of IL-6 and KIM-1 by 23.5% and 22.6%, respectively, suggesting that this drug improves renal inflammation and decreases ischemia in cells of the PT.

Several clinical trials have proven the protective role of SGLT2i over cardiovascular disease (CVD), finding a lower rate of hospitalization or cardiovascular death due to heart failure [35, 36]. In one study, Díaz-Rodríguez et al. [37] assessed the effect of dapagliflozin on epicardial adipose tissue, finding that this drug reduces the release of proinflammatory chemokines and cellular differentiation in this tissue, suggesting a probable way in which SGLT2i could provide a cardioprotective effect on patients with DM. However, the mechanisms responsible for these beneficial effects are not completely understood yet; several hypotheses have been debated including effects on diuresis/natriuresis [38], blood pressure (BP) [39], cardiac energetic metabolism [40], the inhibition of the Na+/H+ antiporter [41], and anti-inflammatory effects [42], among others [43]. Furthermore, based on the results of many clinical trials, SGLT2i have shown substantial cardiovascular benefits (reduction in the risk of HF with reduced ejection fraction, hospitalizations, or composite cardiovascular deaths) and also reduction of kidney disease progression regardless of diabetes status [44].

Among those suggested mechanisms in the heart, SGLT2i drugs can cause attenuation of cardiac inflammation and fibrooxidative stress reduction, ventricular and arterial stiffness reduction, and improved endothelial dysfunction and blood pressure [45]. These mechanisms lead to cardiovascular benefits through reduction in left ventricular (LV) preload and afterload, leading to improved systolic and diastolic functions with reduction of LV mass. These effects could lead to myocardial energy optimization in the form of cardiac ketones and increase cardiac output, heart rate, oxygen consumption, and coronary flow through increased glucagon levels [46].

### 2.2. Effect of SGLT2i on Obesity and Adipose Tissue Inflammation

There is clear evidence of the use of SGLT2i and its impact on obesity, with studies that show an average weight loss of 2 kg, being the most accentuated effect over the first weeks of treatment and modest starting on week 24 [47, 48]. Indeed, studies have reported that SGLT2i treatment in patients with obesity and diabetes reduces total body weight, reducing visceral and/or subcutaneous adipose tissue [49, 50]. These effects are found without affecting bone mineral density or markers of bone turnover (Table 1) [47].

Regarding the mechanisms that favor weight loss, it has been proposed that the decrease in serum glucose due to the chronic use of SGLT2i can also promote a change in the insulin/glucagon ratio, where the proportion of glucagon increases favoring the process of lipolysis and lipid oxidation, while insulin decreases, enhancing the production of endogenous glucose from amino acids (AA), favoring even further the process of lipolysis [51, 52]. Nevertheless, these studies have shown that weight loss tends to be lower than expected, and this is considered to happen due to the compensatory increases in caloric intake (compensatory hyperphagia) that exceed the estimated caloric intake. This could be why after some time taking SGLT2i, weight loss can be attenuated. However, this compensatory mechanism is not still completely elucidated [53, 54]. On the other hand, it has been proposed that the weight-reducing effect of SGLT2i is not absolute to all individuals, and it can vary depending on certain factors such as body mass index (BMI) [55], the presence of genetic polymorphisms [56], the time with T2DM [57], kidney function [58], and the concomitant use of other drugs [59, 60].

SGLT2i drugs also have a role in the mitigation of inflammation induced by obesity and insulin resistance (IR). In obesity, the accumulation of ectopic fat can induce an innate immune response mediated mainly by the recruitment of T cells and macrophages in different tissues [61]. Thus, adipose tissue macrophages (ATMs) increase their polarization as M1-type macrophages (activated in the classic way/proinflammatory) in response to oxidative stress (which increases the levels of free fatty acids). Also, the M1 macrophages are capable of releasing proinflammatory cytokines, including TNF-*α* and IL-6, which via the activation of the JNK, MAPK, and IKKB kinases trigger the phosphorylation of the insulin receptor substrate proteins, directly contributing to IR [62]. Additionally, the activation of Th1 and CD8+ T cells also promotes the recruitment of M1 macrophages in adipose tissue, which generates a maladaptive feedback mechanism, generating more inflammation and favoring IR [63]. Studies have demonstrated that SGLT2i reduce the recruitment and accumulation of T cells and M1 macrophages while increasing the polarization of M2 macrophages (activated alternatively/anti-inflammatory) which release anti-inflammatory cytokines such IL-4, IL-13, and IL-33 that help the ATMs in reducing inflammation and avoiding the progression of IR [64].

On the other hand, M2 macrophages seem to favor white adipose tissue (WAT) browning. According to studies performed in mice that are exposed to cold [65], M2 macrophages release cytokines that activate *β*-adrenergic receptors in adipocytes, which induce thermogenesis, promoting the expression of uncoupling proteins (UCP), specifically UCP1. This is the main isoform expressed in brown adipose tissue (BAT). In contrast, in the presence of inflammation, it has been reported that proinflammatory cytokines activate the JNK protein pathway, favoring the phosphorylation of interferon regulatory factor 3 (IRF3), which leads to a reduction in the expression of UCP1 and, therefore, contributes to the decrease in WAT browning [66].

While WAT comprises a group of unilocular adipocytes related to energy storage, BAT comprises multilocular adipocytes that are rich in mitochondria and are implicated in energy expenditure. These processes are modulated by immune cells, whose pro- or anti-inflammatory phenotype varies according to the microenvironment [67]. It is important to highlight that due to its characteristics, WAT is more prone than BAT to develop inflammation in conditions of metabolic stress [68]. Little is known about the mechanisms implicated in the chronic inflammation of BAT, but its thermogenic activity and browning seem to be disturbed during this process. One of the mechanisms proposed to explain this phenomenon is the chronic activation of the NF-kB pathway, the subsequent reduction in cAMP synthesis, and the increase in norepinephrine remotion by sympathetic neuron-associated macrophages [69]. Furthermore, chronic inflammation has been associated with a change in the composition of immune cells in BAT, where M1 macrophages promote its whitening [67, 68].

Recently, a preclinical study with dapagliflozin reported a significant increase in tyrosine-hydroxylase expression and a high tendency of norepinephrine in WAT of BPH/2J mice suggesting a heightened activity of the sympathetic nervous system (SNS), which has been associated with the browning of WAT. Nevertheless, the authors did not find a significant increase in the mRNA levels of UPC1 and its regulator nor a decrease in IL-6 or TNF-*α* after two weeks in treatment [70]. In contrast, a preclinical study with empagliflozin evidenced that its chronic use (16 weeks) decreased the accumulation of lipids in the BAT and increased energy expenditure which was associated with higher levels of UCP1 both in BAT and in WAT, suggesting that SGLT2i not only participate in the browning of WAT but also induce the activation of BAT. However, these findings were obtained only with high (10 mg/kg) and not low (3 mg/kg) concentrations of the drug, insinuating that the said effects are dose-dependent [64].

Besides, empagliflozin administration resulted in a significant decrease of TNF-*α* mRNA levels and an increase of IL-10 in the WAT [64]. Curiously, it has been suggested that IL-10 could also have a role in the process of WAT browning. Specifically, in a study with mutant *Fas* mice, which have a thin phenotype and express high levels of IL-4 and IL-10, it was found that they have a greater browning response when exposed to cold than wild mice [71]. Meanwhile, TNF-*α* has been proven to suppress adiponectin expression in *in vitro* studies [72]. Adiponectin is an adipokine whose levels are decreased in obese individuals [73] and has anti-inflammatory properties when it interacts with its receptors (AdipoR1 and AdipoR2), favoring the Akt-eNOS-dependent phosphorylation of AMPK and the inhibition of PKA-dependent NF-kB [74–76]. As previously mentioned, the microenvironment can regulate the cell's energetic homeostasis, but in order for the adipocyte differentiation and/or remodeling processes to occur, adequate mitochondrial functioning is necessary [77, 78–80]. According to one study, canagliflozin can upregulate the expression of different mitochondrial genes such as PGC-1*α*, NRF1, TFAM, COX5b, CPT1b, and ACADM, increasing energy dissipation both *in vivo* and *in vitro.* The said increase in mitochondrial biogenesis and function, as well as in fatty acid oxidation, was partly attributed to the dose-dependent increased expression of PPAR*α* [81]. Hence, SGLT2i could promote adequate mitochondrial function and WAT browning, as well as the activation of BAT, via multiple mechanisms that influence the attenuation of inflammation in adipose tissue associated with obesity [82]. Finally, the beneficial effects of these drugs as a therapeutic target to manage inflammation in patients with diabetic kidney disease are promising.

## 3. GLP-1RA: Beyond Its Incretin-Mimetic Effect

After meals especially that food rich in fats and carbohydrates, intestinal cells release a set of hormones that favor insulin release. This phenomenon is known as the “incretin effect” [83]. One of the main hormones within this group is GLP-1. It is mainly released by L cells in the colon and distal ileum [84]. It is capable of reducing serum glucose concentrations by stimulating the glucose-dependent release of insulin, by inhibiting the hypersecretion of glucagon (except during periods of hypoglycemia), and by promoting fullness and slowing intestinal motility, having a glucose-reducing effect without triggering hypoglycemia [85, 86].

Consequently, it was proposed that the stimulation of GLP-1 receptors (GLP-1R) could be a possible therapeutic target in the management of T2DM. However, it must be highlighted that GLP-1 is quickly degraded and inactivated by the enzyme dipeptidyl peptidase-4 (DPP-4), having a half-life of just 2 minutes [87, 88]. Hence, it is necessary to develop compounds capable of mimicking the physiological functions of GLP-1 while being resistant to the action of DPP-4. In this context, exenatide was the first GLP-1 agonist approved by the FDA to treat T2DM. Since then, other compounds have been developed that share the mechanism of action but differ in their structure (based on Exedin-4 or human GLP-1) and half-life (short and long). Each one has its particular indications and advantages [89].

### 3.1. Effect of GLP-1RA on Cardiometabolic and Renal Diseases

To date, there is little evidence about the uses of GLP-1RA with exclusively renal outcomes. For this reason, and indirectly, studies focused on cardiovascular targets providing information on the renal impact of these drugs (Figure 1) [90, 91].

**Figure 3:**
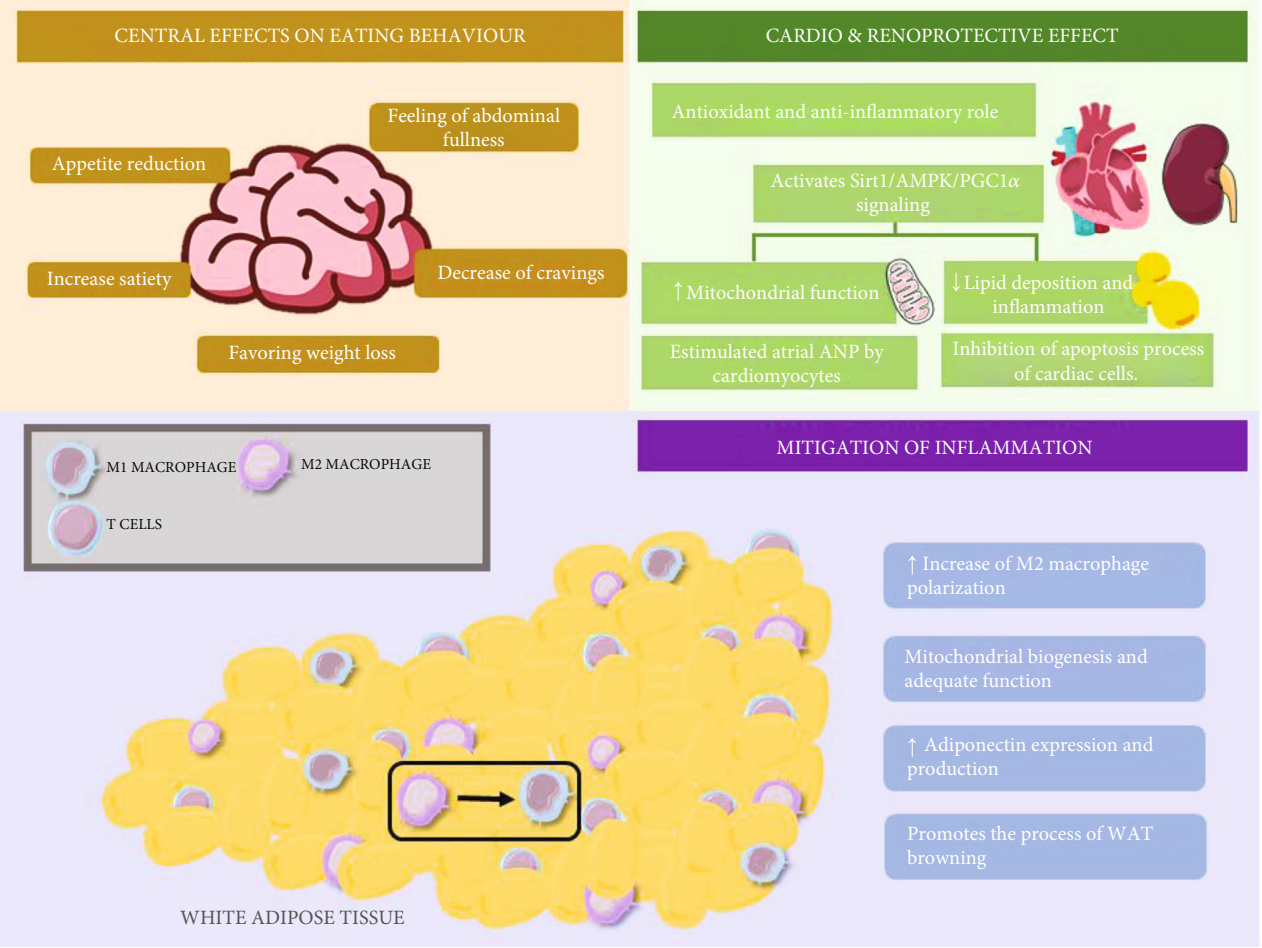
Role of GLP-1RA in obesity, adipose tissue inflammation, and cardiac and kidney disease. In obesity, glucagon-like peptide 1 agonist (GLP-1RA) effects are related to its capacity to penetrate the blood-brain barrier and the presence of the glucagon-like peptide 1 receptor (GLP-1R) in different brain regions, resulting in appetite reduction, increase in satiety, and abdominal fullness and a decrease in food cravings. This, in turn, favors a weight loss of 2 to 8 kg on average. In the kidneys, an antioxidant and anti-inflammatory role has been reported. Via the activation of the Sirt1/AMPK/PGC1*α* signaling pathway, it partially restores the function of renal mitochondria and decreases lipid deposition and inflammation in the kidneys. Lastly, its role has been proven in the mitigation of inflammation since it favors macrophage polarization and adiponectin production while promoting mitochondrial biogenesis and adequate function, as well as adipose tissue browning. GLP-1RA: glucagon-like peptide 1 agonists; GLP-1R: glucagon-like peptide 1 receptor; SIRT1: sirtuin 1; AMPK: AMP-activated protein kinase; PGC1*α*: Pparg coactivator 1 alpha; WAT: white adipose tissue; ANP: atrial natriuretic peptide.

A meta-analysis analyzed a cohort of 56,004 subjects in treatment with GLP-1RA (lixisenatide, liraglutide, semaglutide, exenatide, albiglutide, dulaglutide, or oral semaglutide). This study evidenced that these drugs reduce the incidence of cardiovascular events in patients with diabetes with prior CVD or with risk factors. They also decrease the development of macroalbuminuria, to the detriment of GFR, the progression to end-stage kidney disease, and the mortality rates for renal causes [92]. Similar results were reported by other randomized trials and meta-analyses, which included studies with cardiorenal outcomes of GLP-1RA versus placebo [93] or versus SGLT2i [94, 95] (Table 2). Thus, cardioprotective and renoprotective effects could be an indirect result of the role of GLP-1RA over classic renal and cardiovascular risk factors, such as glycemic control, weight loss [96, 97], blood pressure [98], and decrease of serum LDL-cholesterol and triacylglycerides [99], and even the modulation of the intestinal microbiota (Figure 3) [100, 101].

Furthermore, it has been demonstrated that GLP-1RA can induce natriuresis by inhibiting the action of the sodium-hydrogen antiporter 3 (NHE3) present in the microvilli of the PT, restoring the tubular-glomerular intercommunication. This reduces intraglomerular pressure, hyperfiltration, and the activation of the renin-angiotensin system [102]. Meanwhile, preclinical studies reported an increase in the release of atrial natriuretic peptide (ANP) by cardiomyocytes after the administration of liraglutide [103] and of the signaling of the *β*catenin transcription regulator, involved in the apoptosis process of cardiac cells due to the accumulation of intracellular lipids when using GLP-1RA [104]. These findings have allowed proposing direct protective mechanisms, in addition to the reduction of renal hypoxia, oxidative stress, and local and systemic inflammation [105, 106]. Thus, the majority of data prompt the question of whether GLP-1RA has a class effect. Human GLP-1RA have demonstrated cardiovascular morbidity and mortality reduction and improve the risk of HF events in patients with T2DM, leading to trials testing their efficacy/safety in HF regardless of T2DM [107]. The first reported study [108] was conducted in patients with a recent history of acute coronary syndrome and T2DM, confirming the noninferiority of lixisenatide for three-point major adverse cardiovascular events.

In the kidney, GLP-1RA have also demonstrated an antioxidant and anti-inflammatory role [109]. In this sense, models in mice with induced obesity and changes in renal functions evidence that treatment with liraglutide activates the Sirt1/AMPK/PGC1*α* signaling pathway, which partially restored the function of renal mitochondria and decreased lipid deposition and renal inflammation. This resulted in decreased lipid and energy metabolism disorders in the renal tissue [110].

### 3.2. GLP-1RA in Obesity and Adipose Tissue Inflammation

Aside from the success obtained in glycemic control, GLP-1RA have also been proven to exert a robust effect on weight loss in patients with T2DM, achieving an average reduction of 2 to 8 kg [96, 111], which has allowed to evaluate GLP-1RA as an attractive pharmacological alternative for obese patients. Such is the case of semaglutide whose clinical trials have reported great efficacy in weight loss in patients with T2DM and is being evaluated for its use in patients with obesity and without DM [112]. On the other hand, liraglutide has already been approved by FDA for the pharmacological treatment of obesity in higher doses than those used for DM [113].

This weight loss is attributed to the effects of appetite reduction, increase in satiety and feeling of abdominal fullness, and the decrease of food cravings reported with the use of several GLP-1RA [114, 115]. The mechanism responsible for these effects seems to be associated with the ease with which GLP-1RA can penetrate the blood-brain barrier and with the presence of GLP-1R in certain areas of the brain (highlighting the insula, the amygdala, the putamen, and the orbitofrontal cortex) that are involved in appetite control and ingestion of foods [116, 117] and that are more active in obese subjects [118]. In this sense, studies have shown that the binding of GLP-1RA to GLP-1R in these areas of the brain, along with its subsequent activation, seems to be involved in the suppression of certain metabolic pathways, including brain glycolysis, signaling of protein kinase A (PKA), AMP-activated protein kinase (AMPK), protein kinase B (PKB), and the mechanistic Target of Rapamycin (mTOR), promoting a reduction in caloric intake, which results in weight loss [119].

Additionally, some factors seem to influence the slimming response of GLP-1RA, including sex and BMI. In one study, liraglutide elicited a greater weight loss in women than in men and a lower weight loss in patients with obesity and a BMI > 40 kg/m^2^ [96] as well as in those with genetic polymorphisms of GLP-1R or other components of the signaling pathway [120, 121]. Pharmacokinetics also have a role since liraglutide and semaglutide have greater efficacy for weight loss [122, 123] while others such as albiglutide have a particularly lower efficacy for weight loss [124].

Currently, there is scarce evidence of the inhibitory effect of GLP-1RA on systemic or adipose tissue inflammation in patients with DM (Table 2). Within this context, a placebo-controlled trial in patients with T1DM that were treated with liraglutide or placebo reported that the use of this drug resulted in a decrease in the levels of IL-6, IL-8, IL-10, and INF-*γ* after the 26 weeks of treatment, although this decrease was only significant for IL-6 [125]. Similarly, another clinical study performed in patients with T2DM found that treatment with liraglutide for 6 weeks decreased the expression of inflammatory markers such as TNF-*α*, TLR2, TLR4, and ceruloplasmin [126]. Izaguirre et al. [2] reported that the use of exendin-4 upregulated the mRNA levels of *ADIPOQ* and downregulated the expression of IL-1B, IL-6, IL-8, and TNF genes in the visceral adipocytes of patients with T2DM.

Several *in vitro* studies have delved into the effects of GLP-1RA on adipose tissue and their potential benefit as anti-inflammatory agents. As previously mentioned, the infiltration of M1 macrophages in adipose tissue plays a major role in the low-grade inflammation associated with obesity. It stimulates the release of high concentrations of IL-6 and TNF-*α*, which along with the increase of MCP-1 in this tissue, contributes to greater infiltration of macrophages in the adipocytes, causing an inhibition of the release of adiponectin and contributing to IR in adipose tissue [127]. Currently, it is known that some of the main transcription factors involved in macrophage polarization are the members of the STAT family. Among them, STAT1, which is activated in response to M1 propolarization cytokines (i.e., INF-*γ*), and STAT3 and STAT6 (i.e., IL-4 and IL-10), which are activated by M2 propolarization signals [128, 129]. Studies reported that GLP-1RA are capable of modulating the polarization of MAT towards the M2 phenotype via STAT3, achieving a drastic reduction in the expression of IL-6 and MCP-1, thus reverting the inhibition of the synthesis of adiponectin by M1 macrophages [130, 131].

Besides reverting the inhibitory action of M1, GLP-1RA also favor the expression of adiponectin via GLP-1R. Specifically, exendin-4 has been proven to increase the release of this hormone in 3T3-L1 adipocytes via the GLP-1R/PKA pathway and decrease the levels of proinflammatory adipokines [131]. Furthermore, *in vivo* and *in vitro* studies in 3T3-L1 cells reveal that GLP-1RA modulate the signaling pathway dependent on soluble guanylate cyclase (sGC), exerting a beneficial effect over mitochondrial biogenesis where there is an increase in the expression of BAT genes and mitochondrial markers, which promotes the process of browning of WAT [132]. The nitric oxide (NO) and sGC signaling cascade is the main focus of various pharmacological interventions to improve the inflammatory state in patients with CKD (ClinicalTrials.gov Identifier: NCT04507061). In this regard, the differential expression of enzyme isoforms in various cells and tissues, which mediate cellular effects, also has a direct impact on kidney function. The signaling cascade is currently targeted at the level of cGMP production (nitrates, sGC stimulators, and sGC activators) and cGMP degradation (PDE5 and PDE9 inhibitors) [133].

Lastly, GLP-1RA can modulate the accumulation of fatty acids in BAT and WAT via the brain-adipocyte axis. Particularly, the administration of GLP-1RA appears to decrease the hypothalamic phosphorylation of AMPK and increase sympathetic activity, favoring the process of thermogenesis and browning of BAT while increasing the expression of PG1*α*/UCP1 and promoting mitochondrial activity and the expression of genes involved in fatty acid metabolism [134]. GLP-1RA seem to contribute to mitigating inflammation in adipose tissue associated with obesity by polarizing macrophages, producing adiponectin, and promoting mitochondrial biogenesis and adequate function. Hence, it is vital to consider the association that obesity and an inflammatory state have with cardiometabolic and renal diseases. Moreover, the effect that GLP-1RA have over obesity and inflammation in adipose tissue makes them a promising therapeutic strategy in patients with diabetic complications such as CKD and HF.

## 4. Effect of SGLT2i and GLP-1RA in Cell Senescence

It has been described that the microenvironment elicited by T2DM and obesity is favorable for the development and accumulation of senescent cells. Currently, *in vivo* and *in vitro* studies have reported that hyperglycemia *per se* is capable of accelerating the process of senescence in endothelial cells, preadipocytes, fibroblasts, and mesangial cells, among others. Probably, hyperglycemia accelerates this process by promoting mitochondrial dysfunction, which increases the production of reactive oxygen species (ROS), the accumulation of advanced glycation end-products (AGEs), and marked damage of DNA [135–137]. Similarly, it has been reported that ceramides, whose synthesis is upregulated in obesity and T2DM, induce the expression of senescence markers in endothelial cells and fibroblasts [138, 139]. Moreover, low-grade inflammation present in these diseases is also capable of promoting cellular senescence [140].

However, to his point, one of the most studied accelerating factors of cellular senescence in T2DM, and the one that has been given the leading role in this process, is hyperinsulinemia. High concentrations of this hormone can activate the insulin-like growth factor 1 (IGF-1) receptor, promoting cellular senescence via several pathways dependent and independent of inflammation and oxidative stress. Some of them are ROS-p53, PI3K/p53-p21, SIRT1/p53, and ERK/p53 [140, 141]. Thus, it is necessary to maintain adequate serum levels of glucose and insulin to antagonize premature cellular senescence in T2DM. This necessity stems from the fact that even if these cells are incapable of dividing, they remain metabolically active, releasing a high amount of proinflammatory cytokines, chemokines, and growth factors capable of contributing to the dysfunction and damage of other tissues, favoring the onset of complications and generating a pathological feedback cycle [142, 143].

Currently, there are few studies focused on determining the role of SGLT2i in cellular senescence. Madonna et al. [144] reported that empagliflozin suppressed the senescence induced by hyperglycemia in cultures of murine cardiac stromal cells, reverting the downregulation of the PI3K/Akt pathway, which regulates the expression and activity of several targets implicated in the processes of apoptosis and proliferation. They provided evidence of the *in vivo* antifibrotic and antisenescence role of empagliflozin. However, due to the lack of evidence supporting the expression of SGLT2 receptors in murine cardiac stromal cells and murine cardiomyocytes, the authors propose that these effects are not connected to the effects of SGLT2 on the heart and they remain uncertain.

On the other hand, Sugizaki et al. [141] reported that the treatment with SGLT2i attenuated inflammation, oxidative stress, and cellular senescence, particularly in visceral WAT, and antagonized the endothelial dysfunction in db/db mice. They also observed that serum insulin levels remained relatively stable, just like the mass of beta cells, possibly due to the loss of glucotoxicity via the increase of glucose urinary excretion. These results deferred from the group of mice treated with insulin, who showed a reduction in serum glucose and an attenuation in oxidative stress and inflammation, but a higher expression in senescence markers, which backs up that hyperinsulinemia is the main factor in cellular senescence in this case. Regarding diabetic nephropathy, Kitada et al. [145] reported that hyperglycemia, but not hyperinsulinemia, induced senescence in kidney cells of mice in earlier stages of T1DM. There was an increase in the levels of p21 renal RNA, whose silencing suppressed the process of senescence induced by hyperglycemia in cultures of human primary renal proximal tubule cells (HPTCs). These results suggest that hyperglycemia causes senescence in the proximal tubule cells via a p21-dependent mechanism. The decrease of p21 was attributed to the reduction in the expression of SIRT1 and NAMPT (a regulator of the activation of SIRT1) and therefore the activity of p53, in charge of regulating the transcription of p21 [145].

It is important to highlight that the increase in p21 presented mainly in the S1 and S2 segments of the PT, where SGLT2 carry out their function, but not in other segments. Kitada et al. continued to evaluate if senescence due to high glucose levels was due to an SGLT2-dependent mechanism in cultures of HPTCs, proving that the hyperreabsorption of glucose via SGLT2 contributes to the said process. When studying these results, it must be taken into consideration that this study was carried out in an animal T1DM model where there is normal insulin sensitivity, unlike T2DM, where there may also be other implicated mechanisms dependent on the action of insulin. It would be interesting to delve into the possible effects of SGLT2i on the process of renal senescence.

Oeseburg et al. [146] reported that GLP-1RA had a protective effect over ROS-induced senescence in human umbilical vein endothelial cells (HUVECs), associating this phenomenon to the reduction in DNA damage. In contrast to what was expected, this effect was independent of the PI3K/Akt pathway and NO synthesis. It was the result of the Akt-dependent activation of cAMP, mediated by GLP-1R. This seems to activate defense mechanisms against oxidative stress. Specifically, it was evidenced that it favored the phosphorylation of the CREB transcription factor and the expression of the HO-1 and NQO defense genes regulated by this factor.

In other studies, Zhao et al. [17] reported that exendin-4 was capable of attenuating the angiotensin II- (Ang II-) induced production of superoxide and the resulting senescence of vascular smooth muscle cells (VSMCs), stimulating their function. These protective effects of exendin-4 appear to depend on the inhibition of Rac1 activation via the PKA-dependent activation of GLP-1R/cAMP and the subsequent reduction of ROS by Nox1. In this sense, exendin-4 restored the proliferation capability of prematurely senescent VSMCs, downregulated p53 and p21, and counteracted the Ang II-induced senescence. Liao et al. [147] reported that GLP-1 reduced mitochondrial dysfunction, ROS production, cellular apoptosis, and senescence induced by high glucose and lipid concentrations in human microvascular endothelial cells (HMECs). The antiapoptotic effect shown by GLP-1 in HMECs was related to the inhibition of the JNK1/2 and p23 pathways, unlike the antisenescence effect that resulted from the Akt inactivation.

Finally, the evidence support the possible mechanisms of GLP-1RA as a promising therapeutic strategy to slow the aging and endothelial dysfunction associated with premature senescence in patients with cardiovascular disease and T2DM [148, 149–151, 105, 152]. However, more studies are needed in this area.

## 5. Conclusions

The current evidence supports the hypothesis that SGLT2i and GLP-1RA drugs have beneficial effects beyond glycemic control in diabetic patients with cardiorenal compromise. These drugs not only have renoprotective, cardioprotective, and slimming properties but also favorably modulate the low-grade systemic inflammation, organ-specific fat accumulation, and adipose tissue dysfunction, as well as slow the senescence process in adipocytes and cardiac and renal cells. This places them as potential therapeutic alternatives in the management of multiple comorbidities in patients with DM with cardiorenal involvement.

## Figures and Tables

**Table 1:** Summary of clinical evidence regarding SGLT2i in cardiovascular, renal, and anthropometric outcomes.

Author (REF)	Methodology	Population	*N*	Outcomes
Toyama et al. [28]	Meta-analysis (27 randomized controlled trials).	Efficacy and safety in patients with T2DM and CKD.	7.363	(1) Reduced the risk of cardiovascular death, nonfatal myocardial infarction or stroke (RR, 0.81; 95% CI, 0.70-0.94), and heart failure (RR, 0.61; 95% CI; 0.48-0.78). (2) Reduced the risk of the composite renal outcomes (HR, 0.71; 95% CI, 0.53-0.95). (3) These agents diminished the annual decline in eGFR slope (placebo-subtracted difference of 1.35 mL/1.73 m^2^/y; 95% CI, 0.78-1.93) and control HbA1c (-0.29; 95% CI, -0.39 to -0.19), blood pressure, body weight, and albuminuria.

Neuen et al. [29]	Meta-analysis (4 randomized, controlled clinical trials).	Effects on major kidney outcomes in patients with T2DM.	38.723	Lowered the risk of dialysis, transplantation, or death due to kidney disease (RR, 0.67; 95% CI, 0.52–0.86; *p* = 0.0019), end-stage kidney disease (0.65; 0.53–0.81; *p* < 0.0001), and acute kidney injury (0.75; 0.66–0.85; *p* < 0.0001).

Bae et al. [27]	Meta-analysis (48 randomized controlled clinical trials).	Effects on individual renal outcomes in patients with T2DM.	58.165	(1) Diminished worsening of nephropathy (RR, 0.73; 95% CI, 0.58 to 0.93; *p* = 0.012); significantly reduced urine albumin-to-creatinine ratio (WMD, −14.64 mg/g; 95% CI, −25.15 to −4.12; *p* = 0.006). (2) These drugs lowered the risk of microalbuminuria (RR, 0.69; 95% CI, 0.49 to 0.97; *p* = 0.032) and macroalbuminuria (RR, 0.49; 95% CI, 0.33 to 0.73; *p* < 0.001).

Heerspink et al. [23]	Randomized, double-blind, placebo-controlled study.	Effects of dapagliflozin on eGFR and death from renal or CV causes in CKD patients, with or without T2DM.	4304	(1) In CKD patients, regardless of the presence or absence of DM, the risk of a composite of a sustained preservation in the eGFR of at least 50%. (2) Progression to end-stage kidney disease or death from renal or cardiovascular causes was significantly lower with dapagliflozin than with placebo.

McMurray et al. [24]	Randomized, placebo-controlled trial.	Effects of dapagliflozin or placebo in addition to recommended therapy on patients with HF (ejection fraction of <40%).	4744	(1) The primary outcome occurred 16.3% in the dapagliflozin group and 21.2% in the placebo group (hazard ratio, 0.74; 95% confidence interval (CI), 0.65 to 0.85; *p* < 0.001). (2) Findings in patients with or without diabetes were similar.

Packer et al. [25]	Randomized, placebo-controlled study.	Effects of empagliflozin or placebo in addition to usual therapy on patients with HF (ejection fraction < 40%).	3730	Empagliflozin reduced the combined risk of cardiovascular death or hospitalization for HF in patients with preserved ejection fraction, regardless of the presence or absence of diabetes.

Bolinder et al. [47]	Randomized, double-blind, placebo-controlled study.	Effects of dapagliflozin on glycemic control and body composition in T2DM.	140	Dapagliflozin lowered HbA1c by -0.3%, weight by -4.54 kg, waist circumference by -5.0 cm, and fat mass by -2.80 kg.

Bouchi et al. [49]	Randomized, controlled, 24-week study.	Effects of intensive exercise and dapagliflozin on body composition in T2DM.	146	(1) Intensive exercise did not significantly reduce fat-free mass after treatment (LSM difference -0.1 kg; 95% CI, -0.5 to 0.4). (2) Dapagliflozin was able to promote the reduction in abdominal fat, seemingly leading to further improvements of hyperglycemia and chronic inflammation.

Bolinder et al. [50]	Randomized, multicenter, double-blind, placebo-controlled 24-week study.	Effects of dapagliflozin on body composition measurements on diabetic patients.	182	(1) Dapagliflozin decreased total body weight (95% CI, -2.84 to -1.31; *p* < 0.0001), waist circumference (95% CI, -2.74 to -0.31; *p* = 0.0143), total body fat mass (95% CI, -2.22 to -0.74; *p* = 0.0001), visceral adipose tissue (95% CI, -448.1 to -68.6; *p* = 0.0084), and subcutaneous adipose tissue (95% CI, -359.7 to -10.1; *p* = 0.0385).

**Table 2:** Summary of clinical evidence regarding GLP-1RA in cardiovascular, renal, and anthropometric outcomes.

Author (REF)	Methodology	Population	*N*	Outcomes
Giugliano et al. [93]	Meta-analysis (7 large-scale CV outcome trials).	Impact of GLP-1RA on cardiorenal variables in patients with T2DM.	56.004	(1) Decreased major CV events by 13% (HR, 0.87; 95% CI, 0.80-0.96; *p* = 0.011) and the risk of CV death by 12% (HR, 0.91 (0.86–0.97)), of nonfatal stroke by 16%, of hospitalization for heart failure by 9%, of all-cause mortality by 11% (HR, 0.89 (0.79–0.99)). (2) Reduced the broad composite kidney outcome by 17% (HR, 0.83 (0.69–1.00)), which was driven by a 24% reduction in macroalbuminuria (HR, 0.76 (0.68–0.86)).

Palmer et al. [95]	Meta-analysis (764 randomized, controlled clinical trials).	Evaluate treatment with SGLT-2i and GLP-1RA in patients with T2DM at varying cardiorenal risks.	421.346	(1) SGLT2i and GLP-1RA reduced all-cause mortality, CV mortality, nonfatal myocardial infarction, and kidney failure. (2) GLP-1RA reduced nonfatal stroke more than SGLT-2i, and SGLT-2i reduced mortality and admission to hospital for heart failure more than GLP-1RA.

Zelniker et al. [94]	Meta-analysis (8 clinical trials).	Benefits of SGLT2i/GLP-1RA in patients with or without atherosclerotic CV disease.	77.242	(1) SGLT2i and GLP-1RA lowered major adverse CV events by 11% (HR, 0.89; 95% CI, 0.83-0.96; *p* = 0.001) and 12% (HR, 0.88; 95% CI, 0.84-0.94; *p* < 0.001). (2) The effect was limited to a 14% reduction in those with established atherosclerotic CV disease (HR, 0.86; 95% CI, 0.80-0.93; *p* = 0.002). (3) SGLT2i (HR, 0.62; 95% CI, 0.58-0.67; *p* < 0.001) and GLP1-RAs (HR, 0.82; 95% CI, 0.75-0.89; *p* < 0.001) decreased the risk of CKD progression including macroalbuminuria. However, only SGLT2i diminished the risk of worsening eGFR, end-stage kidney disease, or renal death (HR, 0.55; 95% CI, 0.48-0.64; *p* < 0.001).

Kristensen et al. [92]	(1) Meta-analysis (7 randomized placebo-controlled trials).	Effects of GLP-1RA on CV outcomes.	56.004	(1) The treatment diminished major adverse CV events by 12% (HR, 0.88; 95% CI, 0.82-0.94; *p* < 0.0001), all-cause mortality by 12% (0.88, 0.83-0.95; *p* = 0.001), and hospital admission for heart failure by 9% (0.91, 0.83-0.99; *p* = 0.028). (2) Decrease the broad composite kidney outcome by 17% (0.83, 0.78-0.89; *p* < 0.0001).

Davies et al. [96]	Randomized, double-blind, placebo-controlled, parallel-group trial.	Efficacy and safety of liraglutide for weight management in diabetic patients.	846	Weight loss was 6.0% with subcutaneous liraglutide (3.0 mg dose) and 4.7% with liraglutide (1.8 mg dose) (estimated difference for liraglutide (3.0 mg) vs. placebo, -4.00% (95% CI, -5.10% to -2.90%); liraglutide (1.8 mg) vs. placebo, -2.71% (95% CI, -4.00% to -1.42%); *p* < 0.001).

Bunck et al. [99]	Randomized, double-blinded, placebo-controlledstudy.	Effects of a 1-year treatment with exenatide or insulin glargine on diabetic patients.	69	Exenatide decreased prandial glucose, triglycerides, apo-B48, calculated VLDL-C, FFA, and MDA (*p* < 0.05).

Brock et al. [125]	Randomized, double-blinded, placebo-controlledtrial.	Evaluated anti-inflammatory properties of liraglutide in diabetic patients.	39	(1) The treatment was associated with weight loss (-3.38 kg; 95% CI, -5.29, -1.48; *p* < 0.001) and a reduction in urine albumin/creatinine ratio (-40.2%; 95% CI, -60.6, -9.5; *p* = 0.02). (2) Significantly decreased interleukin-6 levels (-22.6%; 95% CI, -38.1, -3.2; *p* = 0.025).

## Data Availability

No data were used to support this study.

## Additional Points

*Key Messages*. (i) Current evidence supports the hypothesis that SGLT2i and GLP-1RA have beneficial effects beyond glycemic control in high-risk populations. (ii) These novel drugs not only have cardiorenal protection and slimming properties but also favorably modulate the low-grade inflammation in kidneys, the heart, and adipose tissue. (iii) Other potential effects could be related to slowing the senescence process in adipocytes, myocytes, and renal cells. (iv) Improving the adiposopathy process is a potential therapeutic alternative in the management of multiple comorbidities in patients with cardiometabolic and renal diseases.
